# Comparisons of Treatment for HER2-Positive Breast Cancer between Chinese and International Practice: A Nationwide Multicenter Epidemiological Study from China

**DOI:** 10.1155/2021/6621722

**Published:** 2021-09-15

**Authors:** Yi-Qun Han, Zong-Bi Yi, Pei Yu, Wen-Na Wang, Qu-Chang Ouyang, Min Yan, Xiao-Jia Wang, Xi-Chun Hu, Ze-Fei Jiang, Tao Huang, Zhong-Sheng Tong, Shu-Sen Wang, Yong-Mei Yin, Hui Li, Run-Xiang Yang, Hua-Wei Yang, Yue-E. Teng, Tao Sun, Li Cai, Hong-Yuan Li, Xue-Nong Ouyang, Jian-Jun He, Xin-Lan Liu, Shun-E. Yang, You-Lin Qiao, Jin-Hu Fan, Jia-Yu Wang, Bing-He Xu

**Affiliations:** ^1^Department of Medical Oncology, National Cancer Center/National Clinical Research Center for Cancer/Cancer Hospital, Chinese Academy of Medical Sciences and Peking Union Medical College, Beijing, China; ^2^Department of Cancer Epidemiology, National Cancer Center/National Clinical Research Center for Cancer/Cancer Hospital, Chinese Academy of Medical Sciences and Peking Union Medical College, Beijing, China; ^3^Department of Breast Cancer Medical Oncology, Hunan Cancer Hospital, Changsha, China; ^4^Department of Breast Surgery, Henan Cancer Hospital, Zhengzhou, China; ^5^Department of Medical Oncology, Zhejiang Cancer Hospital, Hangzhou, China; ^6^Department of Medical Oncology, Fudan University Shanghai Cancer Center, Shanghai, China; ^7^Department of Breast Cancer, The Fifth Medical Centre of Chinese PLA General Hospital, Beijing, China; ^8^Department of Breast and Thyroid Surgery, Union Hospital, Tongji Medical College, Huazhong University of Science and Technology, Wuhan, China; ^9^Department of Breast Oncology, Key Laboratory of Breast Cancer Prevention and Therapy, National Clinical Research Center for Cancer, Tianjin Medical University Cancer Institute and Hospital, Tianjin, China; ^10^Department of Medical Oncology, State Key Laboratory of Oncology in South China, Sun Yat-Sen University Cancer Center, Guangzhou, China; ^11^Department of Medical Oncology, The First Affiliated Hospital of Nanjing Medical University, Nanjing, China; ^12^Department of Breast Surgery, Sichuan Province Tumor Hospital, Chengdu, Sichuan, China; ^13^Department of Medical Oncology, Yunnan Cancer Hospital, Kunming Medical University, Kunming, China; ^14^Department of Breast Surgery, Cancer Hospital, Guangxi Medical University, Nanning, Guangxi, China; ^15^Departments of Medical Oncology and Thoracic Surgery, The First Hospital of China Medical University, Shenyang, China; ^16^Department of Medical Oncology, Cancer Hospital of China Medical University, Liaoning Cancer Hospital and Institute, Key Laboratory of Liaoning Breast Cancer Research, Shenyang, China; ^17^The 4th Department of Internal Medical Oncology, Harbin Medical University Cancer Hospital, Harbin, China; ^18^Department of the Endocrine and Breast Surgery, The First Affiliated Hospital of Chongqing Medical University, Chongqing Medical University, Chongqing, China; ^19^Department of Medicine Oncology, Fuzhou General Hospital of Nanjing Military Command, Fuzhou, China; ^20^Department of Breast Surgery, The First Affiliated Hospital of Xi'an Jiaotong University, Xi'an, China; ^21^Department of Oncology, The General Hospital of Ningxia Medical University, Yinchuan, Ningxia, China; ^22^Department of Breast Cancer and Lymphoma, Affiliated Tumor Hospital of Xinjiang Medical University, Urumqi, China

## Abstract

**Objective:**

To better understand the status of medical treatment for human epidermal growth factor receptor 2 (HER2)-positive breast cancer and the differences between the Chinese and the international clinical practice.

**Methods:**

This was a retrospective, nationwide, multicenter, epidemiological study of advanced breast cancer patients from China. Between January 01, 2012, and December 31, 2014, a total of 3649 patients, covering 7 geographic regions and 21 institutions, participated in this series of studies. HER2-positive breast cancer was selected among the group and adopted into this study. In comparison, we summarized the demographics and clinical characteristics of HER2-positive breast cancer from the Surveillance, Epidemiology, and End Results (SEER) database.

**Results:**

A total of 918 patients diagnosed as HER2-positive breast cancer patients were included. The median age at diagnosis was 46 years (ranging, 23 to 78) with a single-peak incidence. The proportions of stages II–IV at diagnosis and distance metastasis in viscera were more than half of the participants. In comparison, the prevalence of estrogen or progesterone receptor-positive expression and luminalB subtype was relatively lower than that of the United States. The receipt of chemotherapy was fairly higher, while the usage of targeted therapy was seriously insufficient. Tumor size was in significantly positive associations with the duration of targeted therapy (Kendall's correlation coefficient = 0.3, *P* < 0.0001), while no prohibitive variables among clinical characteristics were detected.

**Conclusion:**

Our study suggested that HER2-positive breast cancer patients were characterized as a younger trend, a lower prevalence of hormonal receptor (HR)-positive expression, and less accessible to anti-HER2 targeted therapy with insufficient duration over the past few years in China. Concerted efforts should be exerted for promising survival benefits in the future. The trial registration number is https://clinicaltrials.gov/ct2/show/NCT03047889.

## 1. Introduction

Globally, breast cancer is the most frequent cancer and the second leading cancer-related death in females [1]. The estimated cases of female breast cancer were about 1.7 million in 2012 [2], while climbing up to 2.1 million in 2018, which accounted for almost a quarter of women cancers [3]. Around 20–25% of breast cancer is characterized as human epidermal growth factor receptor 2 (HER2) overexpression, which predicts a more aggressive biological behavior, a higher risk of early recurrence and metastasis, and a poor prognosis of patients [4].

Reportedly, although the prevalence of breast cancer is the foremost, cancer-related mortality is the sixth in China [5]. Medical treatment has been greatly optimized over the past years and significantly improves the prognosis of breast cancer patients from China. Howbeit, the estimated populations diagnosed with breast cancer have been continuously climbing, with the estimated cases of 2.5 million in the next decade [6], which is becoming a severe health burden of Chinese populations. The nationwide epidemiological study is an effective way to gain a better understanding of the situation.

In this article, we would analyze the differences between China and the United States, explore the reasons behind, and comprehensively depict the status of medical diagnostics and treatment of HER2-positive breast cancer in Chinese populations.

## 2. Materials and Methods

### 2.1. Study Design and Data Sources

This was a retrospectively, nationwide, multicenter, epidemiological study of female advanced breast cancer between January 01, 2012, and December 31, 2014. A total of 21 institutions, covering 7 geographic regions, were selected for this study. The selected institutions were given random month numbers to make the study operable with a representative selection. Physicians from each institution began to collect the information of inpatients in assigned months which were randomly allocated, and if the number of included patients was less than the predetermined volume, more cases from the neighboring months were reviewed until the total admission conformed to the scheme. January and February were excluded from the randomization due to the Spring Festival in China.

Initially, a total of 3649 patients were included in this study. All data were extracted from the medical chart to the designed case report form (CRF) by trained practitioners. Then, two data processors independently recorded and input the data to the database (Epidata). The finished databases were validated and overall checked in Cancer Institution and Hospital, Chinese Academy of Medical Sciences (CHAMS). Detected inconsistencies would be sent back and revised until the content from the two databases was identical. After that, one of the databases was randomly chosen, received a throughout consistency assessment by practitioners from all participating institutions, and finally underwent a quality control with the proportion of 5% in CHAMS. Ultimately, a total of 2747 inpatients, first diagnosed or treated in this interval, were eligible in this series of investigations, which covered 21 institutions and 7 geographic regions of China. CHAMS, as the primary medical center, was in charge of the overall coordination of this research. This series of the study was approved by the Ethics Committee of CHAMS, and patient consent was not necessary.

This study was conducted based on the proportion of HER2-positive breast cancer patients. In this study, we aimed to comprehensively describe the status of treatment for HER2-positive breast cancer, which comprised neoadjuvant, adjuvant treatment, and therapy of advanced disease. Patients were included if the pre- or postoperative immunohistochemical (IHC) results of HER2 were positive, or borderline with amplification confirmed by fluorescence in situ hybridization (FISH) or chromosome in situ hybridization (CISH). Participants were excluded if (1) hormonal receptor (HR) was unknown and (2) clinicopathological characteristics were missing over 50%. The population demographics and clinicopathological characteristics, including histopathological type, histological grade, TNM staging, and hormonal receptors status, in addition to treatment options, were recorded. The test of HER2 was mainly utilized with the method and procedure recommended by the guideline of HER2 Testing in Breast Cancer [7, 8]. Data extraction was cut off on December 31, 2014.

The Surveillance, Epidemiology and End Results (SEER, https://seer.cancer.gov/) database was searched for the data on HER2-positive breast cancer patients diagnosed between 2012 and 2014. Data were extracted using SEER^*∗*^Stat Software (version 8.3.6, https://seer.cancer.gov/seerstat/) on December 31, 2019. Patients were eligible if (1) they were female and (2) the result of HER2 was positive. These HER2 results were derived from a composite algorithm, which mainly includes IHC, FISH, and CISH (https://seer.cancer.gov/seerstat/databases/ssf/her2-derived). All the classification of breast cancer was according to American Joint Committee on Cancer (AJCC) 7^th^ edition in this study.

### 2.2. Outcome Measures and Statistical Analysis

Advanced breast cancer was defined as breast cancer with irresectable local-regional recurrence or distant metastasis and de novo stage IV breast cancer. HER2-positive expression was regarded as an IHC staining of +++, the Fish ratio of HER2 to CEP17 ≥ 2.0, or the HER2 gene copies per nucleus ≥6.0. Estrogen receptor (ER) and progesterone receptor (PR) positive were considered as >1% staining positive in the testing sample by control. The molecular subtypes of breast cancer comprised luminalA (HR + HER2−), luminalB (HR + HER2+), HER2 enriched (HR-HER2+), and triple-negative breast cancer (TNBC) (HR-HER2−). Disease-free survival (DFS) was defined as the period from the curative treatment to first local-regional recurrence or distant metastasis. Overall survival (OS) was regarded as the time interval from the first diagnosis of breast cancer to the death caused by any reason. The evaluation of clinical efficacy was in accordance with Response Evaluation of Solid Tumors (RECIST) version 1.1. The included patients with advanced breast cancer were systematically discussed about the clinicopathological characteristics and treatment in the (neo)adjuvant and advanced stage, respectively, with the aim of giving a comprehensively comparative analysis with those of the patients from the United States in the entire course of HER2-positive breast cancer.

Comparative analyses of population demographics and clinicopathological characteristics of patients from this study and SEER database were performed using Pearson Chi-square and Fishers' exact probability tests for qualitative data and *t*-test or Wilcoxon rank test for quantitative data on normal and abnormal distribution, respectively. Correlation analysis was performed to assess the associations among clinicopathological variables and therapeutic details. This study was statistically two-sided and analyzed by SPSS version 26.0 (IBM Corporation, Armonk, NY, USA), GraphPad Prism version 8.0 (GraphPad Software, La Jolla, CA, USA), and R software (version 3.6.4).

## 3. Results

### 3.1. Clinical Characteristics

**Figure 1:**
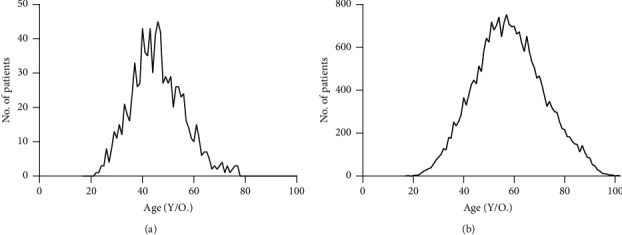
The distribution of age at diagnosis of HER2-positive advanced breast cancer in China (a) and the United States (b) (2012–2014).

A total of 918 patients diagnosed with HER2-positive advanced breast cancer were included in this study (Supplemental Figure 1 and Supplemental Table 1). The median age at diagnosis was 46 years (ranging from 23 to 78) (Figure 1(a)). The percentage of pre- or perimenopausal and postmenopausal was similar with 45.9% and 47.5%, respectively. Pathological stage and histopathological grade tended to be classified as a higher category, of which stages II–IV and grades II-III occupied 68.1% and 55.4%, respectively. As for molecular features, the proportion of hormonal receptors, including ER and PR, was divided equally into positive and negative expression. For molecular phenotype, the percentage of luminalB was slightly higher than that of HER2 enriched with 55.0% and 43.2%, respectively. The de novo stage IV breast cancer holds around 5.9% proportion. With regard to the treatment, the administration of chemotherapy, targeted therapy, endocrine therapy, and radiotherapy was 87.0%, 23.9%, 38.9%, and 43.6%, respectively.

**Figure 2:**
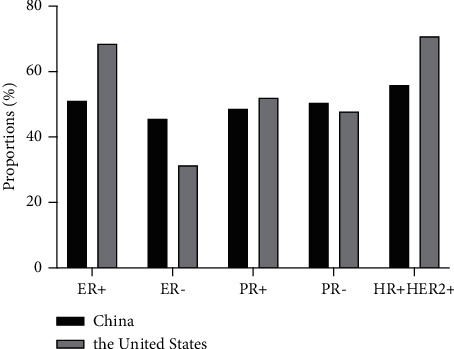
Differences in clinical characteristics and treatment between China and the United States (2012–2014). The columns successively present the percentages of ER status, PR status, and HR+/HER2+ subtype breast cancer.

Meanwhile, data on 24773 HER2-positive advanced breast cancer patients were extracted from the SEER database (Supplemental Table 2). The median age was 58 years (ranging from 17 to 102) (Figure 1(b)). The rate of grades II-III was 94.6%, composing the foremost proportion. The ratios of ER and PR positive expression were 68.5% and 52.0%, which were relatively higher than those of China. Accordingly, HR + HER2+ breast cancer comprised a larger proportion than that of this group population. Comparative results are shown in Table 1 and Figure 2.

### 3.2. (Neo)adjuvant Therapy

In terms of neoadjuvant therapy, a total of 150 patients were included, of whom the diagnostic conclusion of HER2 testing was positive confirmed by puncture (Supplemental Table 3). Anthracyclines were the most frequently used agents in neoadjuvant chemotherapy, while the proportion of taxanes in combination with targeted therapy was fairly low in the populations. The rate of anti-HER2 targeted treatment performance was merely 13.3% with a duration of 5.8 months on average.

The number of patients treated with adjuvant chemotherapy was 799 out of 918, which totals the 87.0% proportion of inclusive participants (Supplemental Table 4). Anthracyclines, taxanes, and the combination were the most common choices when introducing systemic protocol. Approximately half of the patients received radiotherapy with a rate of 47.5%. There were 441 patients administrated with endocrine therapy which was similar to the percentage of HR + HER2+ subtype breast cancer patients at baseline. As for the endocrine agents, selective estrogen receptor modulators (SERMs) were most frequently used, while aromatase inhibitors (AIs) accounted for the second. The practice of combination with ovarian function suppression (OFS) was 3.6%. The percentage of anti-HER2 targeted therapy was 16.4% with an average duration of 8.51 months. Trastuzumab was the most commonly used agent with a percentage of 93.1%.

**Figure 3:**
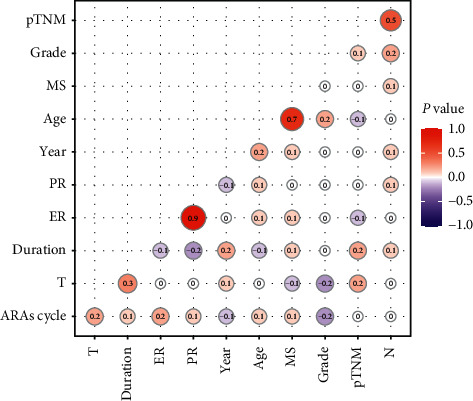
Correlations among the clinical characteristics of HER2-positive breast cancer. Data were presented as Kendall's correlation coefficient (inside the circle) and *P* value (color intensity). MS = menstrual status; PR = progesterone receptor; ER = estrogen receptor; duration = duration of anti-HER2 targeted therapy; T = tumor size; ARAs = anthracyclines.

We also analyzed the multivariables correlations of clinical characteristics and the duration of adjuvant targeted therapy, which included year at diagnosis, age at diagnosis, menstrual status, histopathological grade, tumor size, nodal status, pathological stage, ER and PR status, and the cycles of anthracycline-containing regimens. The positive correlations were detected between the duration and tumor size (Kendall's correlation coefficient, *r* = 0.3, *P* < 0.0001), while no correlations were demonstrated between the duration of anti-HER2 targeted therapy and the additional variables (Figure 3).

### 3.3. Therapy for the Advanced Stage

Among included HER2-positive advanced breast cancer patients, we exhaustively recorded the variables, including progressive status, site of the first progression, rebiopsy performance, the molecular phenotype of metastatic lesions, and treatment details (Supplemental Table 5). In terms of progressive patterns, most patients initially manifested distant metastasis with a percentage of 69.2%, while its coexistence with local recurrence merely occupied 12.9%. The rate of visceral metastasis was relatively higher against nonvisceral metastasis, of which the lung was the most common metastatic site. Most patients received rebiopsy after disease progression, and molecular features varied with the possibility of 43.6%. There were 143 patients who experienced HER2 transition (from positive to negative) with a rate of 30.9%. The secondary molecular phenotype ranges from luminalA to TNBC, with the proportion of 2.6%, 22.5%, 51.6%, and 4.3%, respectively. The DFS of populations was 28.59 months on average, with the 2-year and 3-year DFS rates of 56.9% and 29.4%, respectively.

Most patients were treated with cytotoxic drugs for advanced breast cancer with a percentage of 93.7%. Approximately 476 out of 918 patients received anti-HER2 targeted treatment at an advanced stage, of which the duration on average was 13.46 months. Around 26.8% and 20.5% of participants received anti-HER2 targeted therapy, where trastuzumab and lapatinib took up the most proportion. Synchronously, several inclusive real-world studies also reported the status of trastuzumab-containing therapy. The rates of 1st-line trastuzumab treatment varied from 9.1% to 86.9% in the United States. Just Ray et al. reported that the proportion of trastuzumab-containing treatment at 2nd line was 772 out of 7767 individuals (18.9%) [9].

## 4. Discussion

Population, demographics, and characteristics of HER2-positive breast cancer were different between China and the United States. A younger tendency was shown in patients from China in comparison with those from the United States, with the median age at diagnosis 46 years and 58 years, respectively. Proportions of histopathological grades II-III were both around 95%, whereas the postoperative stages II–IV were approximately 50% of patients from this epidemiologic study. Although the specific stage was not recorded in the SEER database, the foremost percentages of T1-2 and N0-1, with 82.4% and 86.6%, indicated an earlier phase of HER2-positive breast cancer in the United States. The proportion of positively expressed hormonal receptors and luminalB breast cancer was relatively higher among patients in the United States, which reasonably suggested a higher rate of application of endocrine agents. Around 87.0% of patients, owing to the comparatively lower rate of HR-positive breast cancer, received adjuvant chemotherapy in China. Comparatively, patients diagnosed with HER2-positive breast cancer tended to be younger, premenopausal, and HR-negative as well as more accessible to cytotoxic treatment.

In this study, a single peak of diagnosed age was manifested which was in accordance with the tendency of age distribution from patients in the United States. This result was inconsistent with the previous outcome of a double-peak risk (50–54 years and 70–74 years) observed in the last decade from 1998 to 2002 [10]. It has been documented that the age-specific risk of breast cancer varied dramatically over the past decades. Apparent double peaks, of 45–49 years and 70–74 years, were manifested during 2003–2007, while this trend gradually weakened and disappeared in 2012 [11]. The age-specific incidence of breast cancer was considered as associated with multiple socioeconomically and individual factors, such as the educational level, annual earnings, and reproductive patterns in addition to breast cancer screening and awareness. With in-depth exploration in the field of breast cancer, the hypothesis that the differences of tumor characteristics could be the result of intrinsic and molecular features has been gradually established. Intrinsic subtypes tend to vary in ethnics. This hypothesis has been suggested by the higher frequency of luminalB and HER2-enriched breast cancer, among Chinese populations, based on gen- and transcriptomics [12]. Meanwhile, TP53 mutation has been demonstrated a higher occurrence in Asian patients compared with the Western populations [13]. In spite of most conclusions derived from small cohorts or even suffering challenges [14], it could facilitate further scientific research of endogenous heterogeneity among diverse races and ethnicities.

HER2 overexpression, as an independent indicator for the prognosis of HER2-positive breast cancer, is proverbially associated with a higher existence of early recurrence and distant metastasis [4]. In this study, visceral metastasis was shown in more than half of patients, which was in accordance with the general traits of HER2-positive breast cancer. Notably, there was a considerable proportion of metastatic lesions with heterogeneity in molecular subtypes, which luminalA and TNBC also accounted for around 6%. However, just a half of patients, in contrast, received rebiopsy at an advanced stage. More importance should be attached with the rebiopsy performance for advanced breast cancer and further strive for maximum benefits to the prognosis of patients.

Remarkable differences in therapeutics between China and the United States mainly lie in the application of anti-HER2 targeted treatment. This study showed that only a little proportion of HER2-positive breast cancer patients could be accessible to (neo)adjuvant targeted therapy. Besides, the mean duration was limited, fairly short on the recommended continuation of 12 months. Correlation analysis was conducted to clarify the factors associated with the duration of targeted therapy. Kendall's correlation coefficients suggested that tumor size was in positive relation to anti-HER2 continuation. Previous studies have explored the hindering factors of targeted therapy, which included older age, concomitant disease, and ignorance of trastuzumab usage [15]. However, since the studies were mainly conducted in the United States, the results from international, multicenter, large-scale studies are still essential.

Trastuzumab, the first humanized monoclonal antibody against HER2, has been proven to significantly improve the clinical efficacy and prognosis of HER2-overexpressing breast cancer [16–18]. In 1998, the Food and Drug Administration officially approved of listing, yet a 4-year delay of its approval in China [19]. The unaffordable out-of-pocket costs and limited availability seriously prohibited the usage of trastuzumab among Chinese patients. The rate of trastuzumab usage constantly remained low in the last decade, just around 20% even in the high-income areas of China [20]. Moreover, the lack of HER2 standard testing among geographical regions tended to underestimate the HER2-overexpressing rate and further worsen the problems. In this study, the 2-year and 3-year DFS rates were 56.9% and 29.4%, respectively. In contrast, with the 1-year trastuzumab treatment, the 2-year and 3-year DFS rates of HER2-positive breast cancer patients were 87.2% and 78.3%, respectively [21]. It was obviously demonstrated that insufficient targeted therapy caused a poorer prognosis and huge breast cancer burden on patients. Weak awareness of breast cancer screening, the disparities of medical resources, drug insurance policies, and high expenses of novel agents could explain this phenomenon [22, 23]. Continuous efforts from both authorities and researchers have been exerted to resolve this dilemma. The standard operating procedures of HER2 testing were initiated in 2009, updated in 2014, and published in the 2019 edition last year. Also, the charitable program of trastuzumab, sponsored by the Cancer Foundation of China in August 2010 (http://www.cfchina.org.cn/), along with the amendment of drug reimbursement policies, significantly facilitates the application of trastuzumab throughout the nation [19].

The exploration of escalation and deescalation in the field of adjuvant therapy is undergoing for years. The standard timeframe of 12-month application of trastuzumab has been challenged, especially for the low-risk HER2-positive early breast cancer [24, 25]. It enlightens that the short term of targeted therapy could be the optimal choice for not only lower risk of adverse events, especially cardiac toxicity, but also cost-effectiveness considerations. The results from NeoSphere [26], APHINITY [27], and CLEOPATRA trials [28] concertedly optimized the anti-HER2 targeted therapy, which trastuzumab in combination with pertuzumab has become the standard protocol for HER2-positive breast cancer. Trastuzumab emtansine (T-DM1) has managed to be the 2^nd^-line standard treatment option, which was established by the EMILIA trial [29], and could provide significant benefits for HER2-positive breast cancer at the upfront stage [30]. Substantial delays on the time of the application of novel targeted agents in China were obvious. In China, pertuzumab was approved in October 2018 and adopted into medical insurance recently. Besides, T-DM1 was also approved in February 2020. Under these circumstances, the accessibility to anti-HER2 targeted therapy would be definitely updated. With the increasing number of novel targeted agents emerging, tailoring therapy and personalized protocols are bound to play a more promising role in the foreseeable future.

This study did have some substantial limitations. The sample size, of 918 individuals in total, was relatively not enough to represent the general situation of the whole country. Moreover, although the method of ER, PR, and HER2 testing was mainly referred to the national or international guidelines, the inconsistency across remote and underdeveloped regions could not be eliminated, which potentially weaken the reliability of these results. Last, there were a few proportions of data unknown or missing, which could potentially misestimate the real proportions and be less convincing. Nonetheless, considering the fact that this epidemiological study was based on overall coverage of the whole nation in addition to a considerable number of medical institutions, these results could reliably reflect the status of treatment for HER2-positive breast cancer in China.

## 5. Conclusion

In the last decade, patients from China were characterized as a younger trend, a lower prevalence of HR-positive expression, a higher rate of the receipt of chemotherapy, and less access to anti-HER2 targeted therapy with insufficient duration. Nowadays, by virtue of the updated insurance policies, Chinese patients have the tendency to acquire more availability of targeted treatment. With the development of novel agents in addition to the intense exploration of precision medicine, tailoring therapy should be attached to more importance to narrow down the gaps and provide the foremost benefits for breast cancer patients from China in the foreseeable future.

## Figures and Tables

**Table 1:** Clinical characteristics of HER2-positive breast cancer from China and the United States.

Variables	China (*N* = 918)	The United States (*N* = 24773)	*P* value
Age at diagnosis	46 (range, 23 to 78)	58 (range, 17 to 102)	<0.0001
Pathological type (%)			0.079
Ductal or lobular invasive carcinoma	880 (95.9)	24173 (97.6)	
Others	26 (2.8)	600 (2.4)	
Unknown	12 (1.3)	0	
Histopathologic grade (%)			<0.0001
Grade I	15 (1.6)	1234 (5.0)	
Grade II	306 (33.3)	9030 (36.5)	
Grade III	203 (22.1)	14393 (58.1)	
Unknown	394 (42.9)	116 (0.4)	
Molecular features			
ER (%)			<0.0001
Positive	470 (51.1)	16973 (68.5)	
Negative	419 (45.6)	7784 (31.4)	
Unknown	30 (3.3)	16 (0.1)	
PR (%)			<0.0001
Positive	447 (48.7)	12893 (52.0)	
Negative	464 (50.5)	11832 (47.8)	
Unknown	7 (0.8)	48 (0.2)	
Molecular phenotype			<0.0001
HR + HER2+	505 (55.0)	17538 (70.8)	
HR-HER2+	397 (43.2)	7235 (29.2)	
Unknown	16 (1.7)	0	
OS (average, months)	30.16	36.12	

ER, estrogen receptor; PR, progesterone receptor; HR, hormonal receptor; HER2, human epidermal growth factor receptor 2; OS, overall survival.

## Data Availability

The datasets used and/or analyzed during the current study are available from the corresponding author upon reasonable request.

## Supplementary Materials

Supplementary Materials Supplemental Figure 1: flow chart of participants selection. Supplemental Table 1: demographics and clinical characteristics of HER2-positive advanced breast cancer patients (*n* = 918). Supplemental Table 2: characteristics of HER2-positive breast cancer patients in America (*N* = 24773). Supplemental Table 3: neoadjuvant therapy of HER2-positive breast cancer in China (*N* = 150). Supplemental Table 4: adjuvant therapy of HER2-positive breast cancer in China (*N* = 799). Supplemental Table 5: treatment for HER2-positive advanced breast cancer (*N* = 918).
