# Supplementary material for: Comparisons of Treatment for HER2-Positive Breast Cancer between Chinese and International Practice: A Nationwide Multicenter Epidemiological Study from China
Source: J Oncol. 2021 Sep 15;2021:6621722. doi: 10.1155/2021/6621722 (PMC8457988; doi:10.1155/2021/6621722)
Supplement: Supplementary Materials — Supplemental Figure 1: flow chart of participants selection. Supplemental Table 1: demographics and clinical characteristics of HER2-positive advanced breast cancer patients (n = 918). Supplemental Table 2: characteristics of HER2-positive breast cancer patients in America (N = 24773). Supplemental Table 3: neoadjuvant therapy of HER2-positive breast cancer in China (N = 150). Supplemental Table 4: adjuvant therapy of HER2-positive breast cancer in China (N = 799). Supplemental Table 5: treatment for HER2-positive advanced breast cancer (N = 918). [file 6621722.f1.docx]

**Supplemental Figure 1.** Flow chart of participants selection.

**
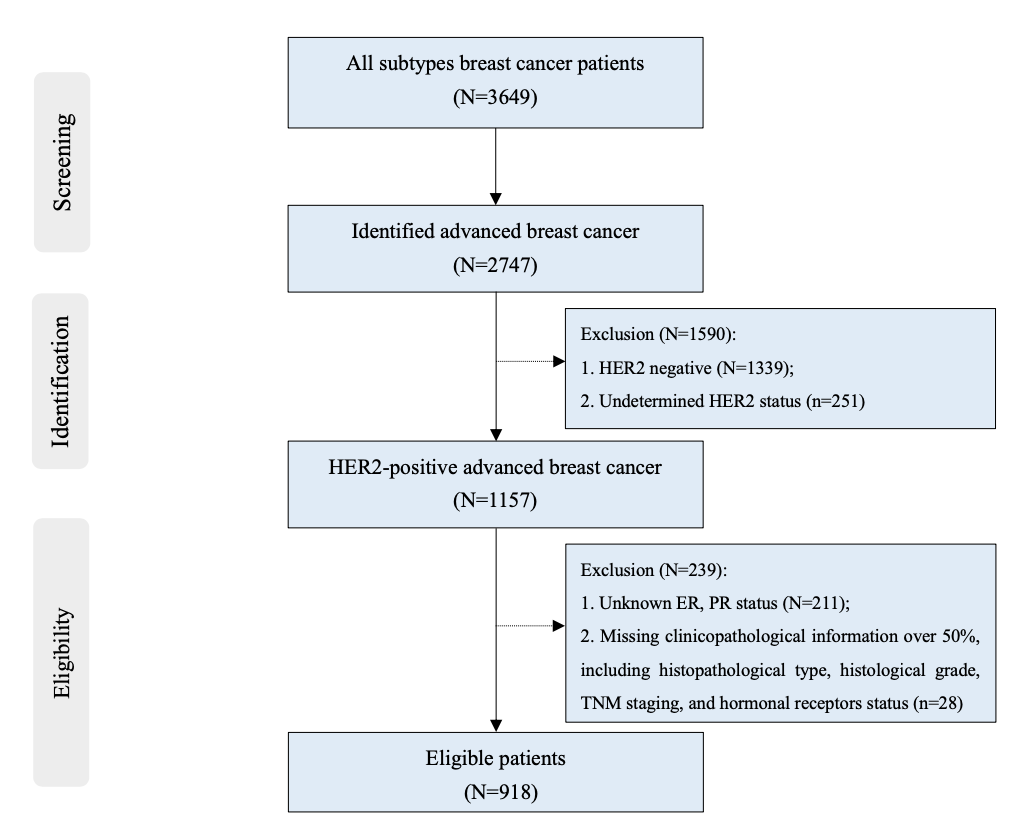
**

**Abbreviations:** HER2, human epidermal growth factor receptor 2; ER, estrogen receptor; PR, progesterone receptor.

**Supplemental Table 1.** Demographics and clinical characteristics of HER2-positive advanced breast cancer patients (n=918).

| Age at diagnosis | 46 (range, 23 to 78) |
| --- | --- |
| Domicile |  |
| Urban | 1654 (46.8) |
| Rural | 1316 (37.3) |
| unknown | 361 (15.9) |
| Marital status |  |
| Single | 88 (2.5) |
| Married | 3367 (95.4) |
| Divorced | 17 (0.5) |
| Others | 23 (0.6) |
| unknown | 36 (1.0) |
| Menstrual status |  |
| Pre- or perimenopausal | 1620 (45.9) |
| Postmenopausal | 1678 (47.5) |
| unknown | 233 (6.6) |
| Surgery |  |
| Mastectomy | 778 (84.7) |
| Segmental mastectomy | 18 (2.0) |
| Conserving surgery | 72 (7.8) |
| unknown | 50 (5.5) |
| Pathologic stage |  |
| Stage I | 61 (6.6) |
| Stage II | 232 (25.3) |
| Stage III | 339 (36.9) |
| Stage IV | 54 (5.9) |
| unknown | 232 (25.3) |
| Pathological type |  |
| Ductal invasive carcinoma | 793 (86.2) |
| Lobular invasive carcinoma | 87 (9.5) |
| Carcinoma in situ | 5 (0.5) |
| Others | 26 (2.8) |
| unknown | 37 (1.0) |
| Histopathologic grade |  |
| Grade I | 15 (1.6) |
| Grade II | 306 (33.3) |
| Grade III | 203 (22.1) |
| unknown | 394 (43.0) |
| Molecular features |  |
| ER (%) |  |
| Positive | 470 (51.1) |
| Negative | 419 (45.6) |
| unknown | 30 (3.3) |
| PR (%) |  |
| Positive | 447 (48.7) |
| Negative | 464 (50.5) |
| unknown | 7 (0.8) |
| Ki67(%) |  |
| <30% | 242 (26.3) |
| 30%-60% | 110 (11.9) |
| >60% | 194 (21.1) |
| unknown | 372 (40.7) |
| Molecular phenotype |  |
| HR+HER2+ | 505 (55.0) |
| HR-HER2+ | 397 (43.2) |
| unknown | 16 (1.8) |
| Chemotherapy |  |
| Yes | 799 (87.0) |
| No | 95 (10.4) |
| unknown | 24 (2.6) |
| Targeted therapy |  |
| Yes | 219 (23.9) |
| No | 678 (73.9) |
| unknown | 21 (2.2) |
| Endocrine therapy |  |
| Yes | 358 (38.9) |
| No | 530 (37.7) |
| unknown | 30 (23.4) |
| Radiotherapy |  |
| Yes | 400 (43.6) |
| No | 484 (52.7) |
| unknown | 34 (3.7) |
| DFS (months) | 28.59 |
| 2-year DFS rate (%) | 56.90% |
| 3-year DFS rate (%) | 29.40% |
| OS (average, months) | 30.16 |
|  |  |

**Abbreviations:** ER, estrogen receptor; PR, progesterone receptor; HR, hormonal receptor; HER2, human epidermal growth factor receptor 2; DFS, disease-free survival; OS, overall survival.

**Supplemental Table 2.** Characteristics of HER2-postive breast cancer patients in America (N=24773).

| Age | 58 (range, 17 to 102) |
| --- | --- |
| Race (%) |  |
| White | 18691 (75.6) |
| Black | 3108 (12.5) |
| Others | 2808 (11.3) |
| unknown | 166 (0.7) |
| Pathological type (%) |  |
| Ductal or lobular | 24173 (97.6) |
| Others | 600 (2.4) |
| unknown | 0 |
| Histopathologic grade (%) |  |
| Grade I | 1234 (5.0) |
| Grade II | 9030 (36.5) |
| Grade III | 14393 (58.1) |
| unknown | 116 (0.4) |
| Subtype |  |
| HR+HER2+ | 17538 (70.8) |
| HR-HER2+ | 7235 (29.2) |
| ER status |  |
| Positive | 16973 (68.5) |
| Negative | 7784 (31.4) |
| unknown | 16 (0.1) |
| PR status |  |
| Positive | 12893 (52.0) |
| Negative | 11832 (47.8) |
| unknown | 48 (0.2) |
| T |  |
| Tis | 22 (0.1) |
| T0 | 25 (0.1) |
| T1 | 11453 (46.2) |
| T2 | 8958 (36.2) |
| T3 | 2023 (8.2) |
| T4 | 1556 (6.8) |
| TX | 590 (2.4) |
| unknown | 4 (0.0) |
| N |  |
| N0 | 14223 (57.5) |
| N1 | 7241 (29.1) |
| N2 | 1731 (7.0) |
| N3 | 1227 (4.9) |
| NX | 351 (1.4) |
| unknown | 0 |
| M |  |
| M0 | 22867 (92.3) |
| M1 | 1906 (7.7) |
| unknown | 0 |
| Radiotherapy |  |
| Yes | 10424 (42.1) |
| No | 14349 (57.9) |
| unknown | 0 |
| Surgery |  |
| Yes | 21876 (88.3) |
| No | 2459 (10.0) |
| unknown | 438 (1.7) |
| Chemotherapy |  |
| Yes | 17991 (72.6) |
| No | 6782 (27.4) |
| unknown | 0 |
| OS (m) | 36.12 (range, 0 to 59) |

**Abbreviations:** ER, estrogen receptor; PR, progesterone receptor; HR, hormonal receptor; HER2, human epidermal growth factor receptor 2; OS, overall survival.

**Supplemental Table 3.** Neoadjuvant therapy of HER2-postive breast cancer in China (N=150).

| Clinical stage |  |
| --- | --- |
| Stage I | 7 (4.7) |
| Stage II | 26 (17.3) |
| Stage III | 81 (54.0) |
| Stage IV | 6 (4.0) |
| unknown | 30 (20.0) |
| Chemotherapy |  |
| Protocol |  |
| Anthracyclines | 130 (86.7) |
| Anthracyclines and taxanes | 89 (58.0) |
| Taxanes and platinum | 5 (3.3) |
| Taxanes, platinum and trastuzumab | 6 (4.0) |
| others | 2 (1.3) |
| unknown | 15 (10.0) |
| Targeted therapy |  |
| Yes | 20 (13.3) |
| Agents |  |
| trastuzumab | 18 (90.0) |
| others | 2 (10.0) |
| Duration (average, months) | 5.77 (range, 1.0 to 21.0) |
| No | 128 (85.3) |
| unknown | 2 (1.3) |

**Supplemental Table 4**. Adjuvant therapy of HER2-postive breast cancer in China (N=799).

| Chemotherapy |  |
| --- | --- |
| Protocol |  |
| Anthracyclines | 578 (72.3) |
| Taxanes | 554 (69.3) |
| Anthracyclines and taxanes | 369 (46.1) |
| Platinum | 24 (3.0) |
| others | 3 (0.3) |
| unknown | 21 (2.6) |
| Targeted therapy |  |
| Yes | 131 (16.4) |
| Agents |  |
| trastuzumab | 122 (93.1) |
| others | 4 (3.1) |
| unknown | 5 (3.8) |
| Duration (average, months) | 8.51 (range, 0 to 24) |
| No | 647 (91.0) |
| unknown | 21 (2.6) |
| Radiotherapy |  |
| Yes | 379 (47.5) |
| No | 398 (49.8) |
| unknown | 22 (2.7) |
| Endocrine therapy |  |
| Yes | 441 (55.2) |
| Agents |  |
| SERMs | 237 (53.7) |
| AIs | 66 (14.9) |
| AIs+OFS | 7 (1.6) |
| SERMs+OFS | 9 (2.0) |
| others | 109 (24.7) |
| unknown | 13 (2.9) |
| No | 341 (42.7) |
| unknown | 17 (2.1) |

**Abbreviations:** SERMs, selective estrogen receptor modulators; AIs, aromatase inhibitors; OFS, ovarian function suppression.

**Supplemental Table 5.** Treatment for HER2-postive advanced breast cancer (N=918).

| Progressive status |  |
| --- | --- |
| Recurrence | 133 (14.5) |
| Metastasis | 635 (69.2) |
| both | 119 (12.9) |
| unknown | 32 (3.4) |
| Site of initial progression |  |
| Bone and soft tissue | 318 (34.6) |
| Liver | 245 (26.7) |
| Lung | 288 (31.3) |
| Brain | 34 (3.7) |
| Visceral metastasis |  |
| Yes | 484 (52.7) |
| No | 414 (45.1) |
| unknown | 20 (2.2) |
| Re-biopsy |  |
| Yes | 463 (50.4) |
| No | 367 (39.9) |
| unknown | 88 (9.7) |
| Molecular status |  |
| consistent | 239 (51.6) |
| inconsistent | 63 (43.6) |
| unknown | 182 (39.3) |
| Molecular phenotype |  |
| LuminalA | 12 (2.6) |
| LuminalB | 104 (22.5) |
| HER2 enriched | 239 (51.6) |
| TNBC | 20 (4.3) |
| unknown | 88 (19.0) |
| HER2 transition (%) | 143 (30.9) |
| Targeted therapy |  |
| Yes | 476 (51.8) |
| 1st-line targeted therapy | 395 |
| Yes | 237 (60.0) |
| Agents |  |
| trastuzumab | 131 (53.3) |
| lapatinib | 41 (17.3) |
| others | 18 (7.6) |
| unknown | 47 (19.8) |
| No | 142 (35.9) |
| unknown | 16 (4.1) |
| 2nd-line targeted therapy | 352 |
| Yes | 188 (60.0) |
| Agents |  |
| trastuzumab | 124 (65.9) |
| lapatinib | 32 (17.1) |
| others | 9 (4.8) |
| unknown | 23 (12.2) |
| No | 142 (35.9) |
| unknown | 16 (4.1) |
| Duration (months) | 13.46 |
| No | 404 (44.0) |
| unknown | 38 (4.2) |
| Chemotherapy |  |
| Yes | 860 (93.7) |
| No | 54 (5.9) |
| unknown | 4 (0.4) |

**Abbreviations:** HER2, human epidermal growth factor receptor 2; TNBC, triple-negative breast cancer.
